# Improving gender equity in access and leadership: regional priorities for change

**Published:** 2025-03-07

**Authors:** Carla Ayres Musa, Elizabeth Kurian, Oteri Eme Okolo, Sareh Safi, Joy Shu'aibu, Minh Anh Tran, Leakhena Or, Valeria Sánchez-Huerta

**Affiliations:** 1 Executive Director: Belize Council for the Visually Impaired, Belize City, Belize.; 2 Chief Executive Officer, Mission for Vision, Mumbai, India.; 3 National Eye Care Coordinator and Director: National Eye Health Programme, Nigeria.; 4 Assistant Professor, Shahid Beheshti University of Medical Sciences, Tehran, Iran.; 5 Country Director, Sightsavers, Abuja, Nigeria.; 6 Optometrist Lead, Department of Ophthalmology - Optometry, Hanoi Medical University, Vietnam.; 7 Chief Deputy, Ophthalmic Outpatients: Preah Ang Doung Hospital, Cambodia.; 8 Asociación para evitar la ceguera en México IAPP, Mexico City, Mexico.

We invited women from several world regions to share the key priorities to achieve gender equity in eye health in their region.

## Sub-Saharan Africa

Women and girls in Africa experience disproportionately low access to eye health services due to a range of social, cultural, and economic barriers. Gender-related barriers, such as prioritising caregiving roles, further hinder women from addressing their eye health needs. To ultimately improve eye care outcomes and foster lasting change, we need to invest in empowering women and girls through gender-transformative approaches.

Experience shows that bridging the gap in access to eye health for women and girls in this region requires meaningful engagement with women, girls and their representative organisations at all levels to ensure that eye health-related interventions reflect their needs and interests. We need policies promoting gender inclusion in eye care programmes and tailored financing options to make eye care more affordable for women. There also needs to be more training opportunities, and an enabling work environment, for female eye care professionals.

## South-East Asia

To promote more female leaders in eye health in Southeast Asia, a multi-pronged approach is needed. In Vietnam, structural challenges like the earlier retirement age (60 years for women versus 65 for men) and extended maternity leave impact career growth.

In Cambodia, and many other South-East Asian countries, despite an increase in female ophthalmologists, optometrists and eye health workers, sociocultural barriers persist. Family responsibilities often take priority over professional development, especially in rural areas.

More efforts are needed to incorporate gender equality into policies and raise women's visibility in leadership. Strategies required to build a supportive environment, which will increase female leadership in eye health across the region include harmonised policies which provide flexible work, awareness campaigns to change cultural norms, and expanded mentorship and training programmes to ensure women are prepared for leadership roles.

## Middle East and North Africa

Priorities that can improve eye health for women and girls vary across countries in the Middle East and North Africa region due to sociodemographic and cultural diversity. In lower-income countries, increasing access to eye care is the main priority. Low family incomes lead to allocating the family budget to basic needs and life-threatening health issues. Improvement is needed in the physical accessibility of affordable and sustainable eye care services for women and girls.

In high- and higher-middle-income countries in this region, community-based awareness and empowerment campaigns focused on women and girls’ eye health is needed to place eye health higher on the priority list for women, their families, and other stakeholders. These should focus on the importance of women's eye health in enhancing the individual and family's quality of life and productivity.

## Latin America

In Latin America, a deeply entrenched culture of ‘machismo’ and systemic inequality exacerbate existing barriers. Women from rural, indigenous, and Afro-descendant communities encounter complex challenges stemming from historical marginalisation, language barriers, and geographic isolation. The region also grapples with one of the highest rates of gender-based violence, including femicide, which jeopardises women's safety and constrains public engagement. Although laws promoting gender equality exist, their enforcement remains weak due to political instability and corruption. Economic disparities, more pronounced than in many other regions, further hinder women's access to resources and opportunities.

Nevertheless, Latin America demonstrates remarkable resilience through grassroots feminist movements like “Ni Una Menos,” (Not One Woman Less) which advocates for policy changes such as the legalisation of abortion in Argentina. To tackle these challenges, culturally tailored solutions are crucial. By harnessing its activist strength and confronting systemic barriers, Latin America can potentially lead significant advancements in gender equity.

## South Asia

Although the region has made significant economic strides, within the predominantly patriarchal societies, progress on gender equity continues to be inadequate. Consequently, girls and women commonly experience stigma and discrimination, with lower access to health, education, employment, and political participation.

A multifaceted, integrated approach is needed to address cultural barriers, promote female leadership, harness innovation, and ensure inclusivity. For example, more supportive workplace policies are required that address the unique challenges women face. Fortunately, within the region we have successful gender-focused models that could be replicated, including the higher uptake of services by women when care is provided by female health workers. Our region also has extensive technological capacity, with telemedicine and mobile health applications offering promising strategies to address some of the barriers experienced by women and girls. Finally, a key priority is to involve communities in discussions about gender equity and health, ensuring that the voices of women and girls are heard and prioritised in the development of health programmes.

